# The Association of Selective Serotonin Reuptake Inhibitors with Delirium in Post-Operative Adults: A Secondary Analysis of a Post-Operative Dataset with Daily Severity of Illness Adjustment

**DOI:** 10.56392/001c.94253

**Published:** 2024-05-23

**Authors:** C. Adrian Austin, Imani Bazemore, Joe Yi, Sarah Glier, Shannon S. Carson

**Affiliations:** 1 University of North Carolina at Chapel Hill

**Keywords:** Delirium, SSRI, Postoperative

## Abstract

**Background:**

Postoperative delirium is a prevalent condition associated with increased mortality, difficulties with physical recovery from surgery and decreased long-term cognitive function, especially in older adults. Currently, there are no direct medical treatments for delirium. We recently found an association between SSRI administration and reduced delirium in a critically ill medical population. We sought to evaluate this association in a surgical population. SSRIs may provide a new treatment option for delirium; further exploration is warranted. We aimed to assess the association between selective serotonin reuptake inhibitors (SSRIs) and delirium in postoperative adults.

**Methods:**

We undertook a secondary analysis of an existing cohort in a large Academic Medical Centre in the Southeast United States. Patients were adults (aged 18–99) requiring at least one night of hospital admission following a scheduled surgery, enrolled from July 2017 to September 2017. Our primary outcome was the incidence of delirium 24 hours after administration of an SSRI. Our exposure variable was any SSRI administration in the preceding 24 hours. We collected data on demographics, SSRI administration, overall severity of illness via the ASA grading system, and daily severity of illness via the Sequential Organ Failure Assessment (SOFA) score from the electronic medical record review.

**Results:**

We collected data on 191 patients (mean age 56.8 years, SD +/− 16.7). One hundred ten (57.6%) were female, and 149 (78%) were White. Most patients, 183 (95.8%), were non-Hispanic. Twenty-eight (14.6%) were prescribed SSRIs at any point during the study period and 35 (18.3%) were delirious on day one. Unadjusted analysis demonstrated that patients receiving SSRIs had OR 1.60 for delirium the next day (p=0.41). After adjusting for age ASA, age, hospital LOS, and SOFA, patients receiving SSRIs had OR 1.44 for next-day delirium (p=0.48).

**Conclusions:**

SSRIs administered in the postoperative period were not associated with delirium on the subsequent day. This finding conflicts with prior results from a critically ill population. The association of SSRIs with delirium requires further investigation.

## BACKGROUND

Delirium, a form of acute brain failure, defined by inattention and a fluctuating course, is highly prevalent in the adult population. Between 4% and 61% of adults requiring elective major surgery will experience postoperative delirium.^1–6^ Patients with delirium have increased risk of adverse outcomes, including increased risk of in-hospital death, one-year mortality, increased costs, and increased risk of long-term cognitive dysfunction.^6–10^

Despite its prevalence and association with poor outcomes, there are no proven effective medical treatments for delirium. One of the primary difficulties with defining a medical treatment for delirium is that our understanding of the pathophysiological cascades associated with delirium is in its infancy. There are numerous hypotheses for the neuropathophysiology of delirium, one of which focuses on neurotransmitter imbalance as a cause.^11^ A large multicentre randomised controlled trial investigated drugs primarily acting on dopaminergic pathways in delirium, finding no role for delirium treatment.^12^ Medications that safely modulate other neurotransmitters in delirium are worthy of investigation. Selective serotonin reuptake inhibitors (SSRIs) are widely prescribed, with the opportunity to assess their potential role in delirium.

Approximately 11% of the U.S. population is on an antidepressant, with SSRIs being the most commonly prescribed drug class.^13^ SSRIs primarily exert antidepressant effects by neuronal remodelling, typically weeks after initiation.^14^ SSRIs, however, do cause acute changes in serotonin levels shortly after their initiation, and this could have possible effects on delirium.^15^ Further, clinical equipoise exists on whether to continue or discontinue SSRIs if patients are prescribed them before an ICU admission during which they become delirious.

In a prior retrospective cohort study, we demonstrated that SSRI administration may be associated with decreased risk of delirium on the same day of administration and at 24 hours in critically ill adults.^16^ However, this work was limited by the inability to adjust for daily severity of illness. To further elucidate the relationship between SSRIs and delirium, we sought to examine the association of SSRIs and delirium incidence in a cohort of postoperative adults that have daily severity of illness measures. Our primary research question is whether SSRI administration is associated with delirium in the subsequent 24 hours after drug administration when adjusted for daily severity of illness.

## METHODS

We conducted a secondary analysis of a postoperative data set.^6^ Participants in the initial study were enrolled from a single academic medical centre (>800) beds in the south-eastern United States. The cohort enrolled 191 adults (18 years and older) patients scheduled for major surgery between July 2017 and September 2017. Major surgery was defined as a non-emergent surgery requiring at least one night of postoperative hospital admission. In addition to demographics, the study collected data on psychoactive medication administration (including SSRIs), daily delirium assessments for the first three days postoperatively, and daily severity of illness via a modified version of the sequential organ failure assessment score (SOFA).^17^ We used the Confusion Assessment Method for the ICU (CAM-ICU) for non-verbal patients and the 3D-CAM for verbal patients.^18,19^ All delirium assessments were performed by research personnel with formal training in delirium assessments.

The University of North Carolina Institutional Review Board Reviewed and approved the study protocol. For the initial study, participants provided verbal informed consent; surrogate consent was obtained if the patients had cognitive impairment. Patients did not receive financial compensation.

Demographic information was obtained via chart review and included age, race, gender, and ethnicity. Medication administration during ICU admission was determined by review of admission documents, medication lists, and preoperative anaesthesia assessment. We specifically looked for selective serotonin reuptake inhibitors (e.g. citalopram, escitalopram, sertraline, fluoxetine, paroxetine, and fluvoxamine). Daily medication administration and dosage during admission was obtained via chart review from the medication administration log for postoperative days 1–3.

To calculate the SOFA score, we collected the lowest SpO2/FiO2 ratio, and the lowest blood pressure reading for each day 1 through 3. Renal disease was assessed by using the creatinine lab value at the time of surgery, and liver disease was assessed by evidence of jaundice or scleral icterus recorded in the physical exam of provider notes those days. We did not include mental status exam in the SOFA given that delirium was our primary outcome. This approach has been used in prior delirium RCTs.^12^

Our primary outcome of interest was the presence of delirium during the day immediately after surgery. Our exposure was SSRI administration on the prior day.

This analysis used logistic regression to observe the association between exposure to SSRI 24 hours prior and delirium the following day. Potential confounding variables were considered a priori based on clinical rationale and/or published literature and included in the models. Covariates selected as potential confounders included age at consent (continuous), hospital length of stay (continuous), preoperative severity of illness as measured by the American Society of Anesthesia preoperative risk score ASA (categorical), and modified SOFA score (continuous). Age was selected a potential confounder as delirium risk increases with age.^20^ Preoperative ASA was selected as patients with a higher baseline comorbidity burden are more likely to develop delirium.^21^ Daily severity of illness as a SOFA score was chosen because patients who are more ill are more likely to develop delirium.^20^ Significance was set at *α*=0.05. We used SAS (version 9.4) for all analyses.

## RESULTS

We collected data on 191 patients. The mean age was 57.38 years (SD +/− 18.9). 110 (57.6%) were female, and 149 (78%) were White. Most patients, 183 (95.8%) were non-Hispanic. 29 (15.2%) were prescribed SSRIs at any point during the study period (Table 1). The mean hospital length of stay was 3 days (SD +/− 3.5) and the mean duration of anaesthesia was 277.5 minutes (SD +/− 16.7 minutes). Thirty-five (18.3%) were delirious on day one. (Table 1).

Unadjusted analysis demonstrated that patients receiving SSRIs had OR 1.60 for delirium on the subsequent day (p=0.41). Analysis adjusted for age, preoperative ASA, and modified SOFA score demonstrated that patients receiving SSRIs OR 1.44 for next-day delirium. This value did not reach statistical significance (p=0.48).

## DISCUSSION

In our secondary analysis of a cohort of postoperative adults, we did not find an association between receipt of SSRIs and the odds of being delirious on the subsequent day. This lack of association remained when adjusted for age, ASA, LOS, and SOFA.

The percentage of patients in our sample receiving SSRIs (14.6%) is higher than the prevalence of antidepressant use prevalence in the general U.S. population (10%).^13,22^ This echoes similar findings from prior work examining SSRIs in the critically ill.^16^ This finding may reflect that patients requiring major surgery have a higher burden of mental health issues compared to the general population.

To our knowledge, this is the first study examining the association of SSRIs and delirium in a postoperative adult cohort. Our findings are important because they conflict with prior work examining SSRIs and delirium in the critically ill. Our group had previously demonstrated an association between SSRIs and decreased rates of delirium in an ICU cohort.^16^

Multiple potential reasons exist for conflicting results from the postoperative compared with critically ill populations. First, the sample size of this current study was smaller than that of the prior study (191 vs 821 patients). Therefore, our current study is likely underpowered to detect a small difference in delirium incidence. Secondly, the current study had a much lower illness severity than prior work. This, coupled with inherent differences in other risk factors for delirium between ICU and postoperative patients, was reflected in a lower incidence of delirium in our current study. This lower severity of illness likely resulted in a much higher threshold for an impact on delirium incidence.

The differences in our study from prior work could also be explained by inherent differences between the insults incurred by critically ill patients and postoperative patients. Stress and inflammatory effects could affect neuropsychoimmunological pathways differently in medically critically ill patients vs surgical patient populations.^23^ Delirium in surgical populations may rely more on physical and localised inflammatory pathways which differentially activate hypothalamic-pituitary-adrenal (HPA) and neurochemical pathways.^24^ In contrast, medical critically ill populations may experience more systemic inflammatory responses. The increased severity of illness and resultant multiple insults associated with it (i.e. hypoxia, hypotension, electrolyte abnormalities, etc.) in critically ill patients may lead to more of a direct insult to the brain compared to surgical insults (minimal prolonged hypotension with immediate anaesthesia intervention). Finally, as delirium is a heterogeneous syndrome, there could be other inherent neurochemical differences in postsurgical vs critically ill populations that result in differential responses to serotonin pathway manipulation. Thus, serotonin pathways may be more favorable for intervention in the ICU population compared to surgical patients.

There has been growing interest in the potential usage of SSRIs as an acute intervention. In particular, the SSRI fluvoxamine has been studied as an intervention for the care of patients with COVID-19. Interestingly, and similar to our current work, studies examining fluvoxamine and COVID-19 have had conflicting results. One large trial found that fluvoxamine resulted in the need for reduction of hospitalisation or emergency care in outpatients with COVID-19.^25^ However, a subsequent trial did not find an association between fluvoxamine and decreased symptom duration or prevention of hospitalisation in outpatients with COVID-19.^26^ These conflicting results may be explained by differences in doses used in the two trials or differences in severity of illness between the two populations sampled.

Given the conflicting results of studies examining SSRIs and important outcomes such as delirium, further research in this field is warranted. SSRIs have a favourable safety profile relative to other medications that have been studied and are used clinically for delirium intervention, such as antipsychotics. A multicentre, retrospective cohort study examining SSRIs and delirium in restricted to critically ill populations is warranted and is the next logical step in this line of research.

This study had a few limitations. The retrospective cohort design raises the potential for unmeasured confounders affecting our observations. Our sample size is smaller than prior work; therefore, it may be underpowered to detect small differences in the effect of SSRIs. Also, the study was conducted at a single academic medical centre in the southeast United States, and results may not be generalisable to other regions or healthcare settings. However, the study enrolled a broad heterogeneous cohort. The CAM could have misclassified patients as delirious when they were not and vice versa. However, all research assessments were performed by trained research personnel with experience in delirium research, which mitigates this risk. Exploring the effect of individual SSRIs on delirium would be interesting for future work but was beyond the scope of this project. Finally, we did not have baseline cognitive function available for this dataset. Cognitive function certainly can affect whether patients are more or less likely to be delirious. However, in this cohort, it is unlikely to be related to whether or not they were prescribed SSRIs. Since it is not related to both the exposure and outcome, we did not feel it reflected a likely source of confounding.

In summary SSRI administration was not associated with a reduced incidence of delirium in the subsequent 24 hours after administration in this postsurgical cohort. Further work examining the relationship between SSRIs and delirium in different populations at high risk for delirium is warranted and may provide avenues for future delirium interventions.

## Figures and Tables

**Table 1.** Demographics and Outcomes

Total Sample n=191	Received SSRI (n=28)	Did not Receive SSRI (n=163)
Age, Yrs, Mean (SD)	57.3 (18.9)	61.5 (22.7)
Sex, n (%)		
Female	22 (79%)	88 (54%)
Male	6 (21%)	74 (45%)
Other	0	1 (1%)
Race		
White	25 (89%)	124 (76%)
Black	2 (7%)	32 (20%)
Other	0	1 (1%)
Ethnicity		
Hispanic/Latino	1 (4%)	1 (1%)
Not Hispanic/Latino	27 (96%)	156 (96%)
Pre-OP ASA, Mean (SD)	3 (1)	3 (1)
No Risk	0	1 (1%)
Low Risk	8 (30%)	54 (34%)
Moderate Risk	16 (59%)	98 (61%)
High Risk	3 (11%)	7 (4%)
Not Expected to Survive	0	0
Surgery Type		
GI	4 (14%)	38 (23%)
OMFS	0	2 (1%)
Cardiac	2 (7%)	7 (4%)
ENT	5 (18%)	9 (5%)
GYN	2 (7%)	11 (7%)
Neurosurgery	3 (11%)	17 (10%)
Ortho	3 (11%)	17 (10%)
Plastics	1 (4%)	8 (5%)
Surg Onc	1 (4%)	7 (4%)
Thoracic	2 (7%)	9 (6%)
Urology	5 (18%)	30 (18%)
Vascular	0	8 (5%)
SOFA, Median (IQR)	2 (2)	2 (3)
		
**Outcomes**		
Hospital Length of Stay, Days, Median (IQR)	3 (3.5)	3 (8.0)
Duration of Anesthesia, minutes, Median (IQR)	277.5 (211.5)	254 (184)
Delirious on Day 1		
Yes	7 (25%)	28 (17%)
No	21 (75%)	135 (83%)

## References

[1] Lundström M, Edlund A, Bucht G, Karlsson S, Gustafson Y. Dementia after delirium in patients with femoral neck fractures. J Am Geriatr Soc. 2003;51(7):1002–1006. doi:10.1046/j.1365-2389.2003.51315.x 12834522

[2] Marcantonio ER, Flacker JM, Michaels M, Resnick NM. Delirium is independently associated with poor functional recovery after hip fracture. J Am Geriatr Soc. 2000;48(6):618–624. doi:10.1111/j.1532-5415.2000.tb04718.x 10855596

[3] Rudolph JL, Inouye SK, Jones RN, Delirium: an independent predictor of functional decline after cardiac surgery. J Am Geriatr Soc. 2010;58(4):643–649. doi:10.1111/j.1532-5415.2010.02762.x 20345866 PMC2856754

[4] Milstein A, Pollack A, Kleinman G, Barak Y. Confusion/delirium following cataract surgery: an incidence study of 1-year duration. Int Psychogeriatr. 2002;14(3):301–306. doi:10.1017/s1041610202008499 12475090

[5] Scholz AF, Oldroyd C, McCarthy K, Quinn TJ, Hewitt J. Systematic review and meta-analysis of risk factors for postoperative delirium among older patients undergoing gastrointestinal surgery. Br J Surg. 2016;103(2):e21–e28. doi:10.1002/bjs.10062 26676760

[6] Austin CA, O’Gorman T, Stern E, Association Between Postoperative Delirium and Long-term Cognitive Function After Major Nonemergent Surgery. JAMA Surg. 2019;154(4):328–334. doi:10.1001/jamasurg.2018.5093 30649138 PMC6484789

[7] McCusker J, Cole M, Abrahamowicz M, Primeau F, Belzile E. Delirium predicts 12-month mortality. Arch Intern Med. 2002;162(4):457–463. doi:10.1001/archinte.162.4.457 11863480

[8] Pandharipande PP, Girard TD, Jackson JC, Long-term cognitive impairment after critical illness. N Engl J Med. 2013;369(14):1306–1316. doi:10.1056/nejmoa1301372 24088092 PMC3922401

[9] Leslie DL, Marcantonio ER, Zhang Y, Leo-Summers L, Inouye SK. One-year health care costs associated with delirium in the elderly population. Arch Intern Med. 2008;168(1):27–32. doi:10.1001/archinternmed.2007.4 18195192 PMC4559525

[10] Pandharipande PP, Ely EW, Arora RC, The intensive care delirium research agenda: a multinational, interprofessional perspective. Intensive Care Med. 2017;43(9):1329–1339. doi:10.1007/s00134-017-4860-7 28612089 PMC5709210

[11] Maldonado JR. Delirium pathophysiology: An updated hypothesis of the etiology of acute brain failure. Int J Geriatr Psychiatry. 2018;33(11):1428–1457. doi:10.1002/gps.4823 29278283

[12] Girard TD, Exline MC, Carson SS, Haloperidol and Ziprasidone for Treatment of Delirium in Critical Illness. N Engl J Med. 2018;379(26):2506–2516. doi:10.1056/nejmoa1808217 30346242 PMC6364999

[13] CDC. Antidepressant Use in Persons Aged 12 and Over: United States, 2005–2008. Published 2011. Accessed September 15, 2015. http://www.cdc.gov/nchs/data/databriefs/db76.htm

[14] Krishnan V, Nestler EJ. The molecular neurobiology of depression. Nature. 2008;455(7215):894–902. doi:10.1038/nature07455 18923511 PMC2721780

[15] Preskorn SH. Clinically relevant pharmacology of selective serotonin reuptake inhibitors. An overview with emphasis on pharmacokinetics and effects on oxidative drug metabolism. Clin Pharmacokinet. 1997;32(Suppl 1):1–21. doi:10.2165/00003088-199700321-00003 9068931

[16] Austin CA, Yi J, Lin FC, The Association of Selective Serotonin Reuptake Inhibitors With Delirium in Critically Ill Adults: A Secondary Analysis of the Bringing to Light the Risk Factors and Incidence of Neuropsychologic Dysfunction in ICU Survivors ICU Study. Crit Care Explor. 2022;4(7):e0740. doi:10.1097/cce.0000000000000740 35923593 PMC9329078

[17] Grissom CK, Brown SM, Kuttler KG, A modified sequential organ failure assessment score for critical care triage. Disaster Med Public Health Prep. 2010;4(4):277–284. doi:10.1001/dmp.2010.40 21149228 PMC3811929

[18] Ely EW, Margolin R, Francis J, Evaluation of delirium in critically ill patients: validation of the Confusion Assessment Method for the Intensive Care Unit (CAM-ICU). Crit Care Med. 2001;29(7):1370–1379. doi:10.1097/00003246-200107000-00012 11445689

[19] Marcantonio ER, Ngo LH, O’Connor M, 3D-CAM: derivation and validation of a 3-minute diagnostic interview for CAM-defined delirium: a cross-sectional diagnostic test study. Ann Intern Med. 2014;161(8):554–561. doi:10.7326/m14-0865 25329203 PMC4319978

[20] Vasilevskis EE, Han JH, Hughes CG, Ely EW. Epidemiology and risk factors for delirium across hospital settings. Best Pract Res Clin Anaesthesiol. 2012;26(3):277–287. doi:10.1016/j.bpa.2012.07.003 23040281 PMC3580997

[21] Marcantonio ER. Delirium in Hospitalized Older Adults. N Engl J Med. 2017;377(15):1456–1466. doi:10.1056/nejmcp1605501 29020579 PMC5706782

[22] Brody D, Gu Q. Antidepressant Use Among Adults: United States, 2015–2018. NCHS Data Brief. 33054926

[23] Cunningham C, Maclullich AM. At the extreme end of the psychoneuroimmunological spectrum: delirium as a maladaptive sickness behaviour response. Brain Behav Immun. 2013;28:1–13. doi:10.1016/j.bbi.2012.07.012 22884900 PMC4157329

[24] McIntosh TK, Bush HL, Yeston NS, Beta-endorphin, cortisol and postoperative delirium: a preliminary report. Psychoneuroendocrinology. 1985;10(3):303–313. doi:10.1016/0306-4530(85)90007-1 2932761

[25] Reis G, Dos Santos Moreira-Silva EA, Silva DCM, Effect of early treatment with fluvoxamine on risk of emergency care and hospitalisation among patients with COVID-19: the TOGETHER randomised, platform clinical trial. Lancet Glob Health. 2022;10(1):e42–e51. doi:10.1016/s2214-109x(21)00448-4 34717820 PMC8550952

[26] McCarthy MW, Naggie S, Boulware DR, Effect of Fluvoxamine vs Placebo on Time to Sustained Recovery in Outpatients With Mild to Moderate COVID-19: A Randomized Clinical Trial. JAMA. 2023;329(4):296–305. doi:10.1001/jama.2022.24100 36633838 PMC9857647

